# Predicting evidence-based treatment sustainment: results from a longitudinal study of the Adolescent-Community Reinforcement Approach

**DOI:** 10.1186/s13012-017-0606-8

**Published:** 2017-06-13

**Authors:** Sarah B. Hunter, Bing Han, Mary E. Slaughter, Susan H. Godley, Bryan R. Garner

**Affiliations:** 1 RAND, 1776 Main Street, Santa Monica, CA 90407-2138 USA; 2 RAND, 4570 Fifth Ave., Suite 600, Pittsburgh, PA 15213 USA; 3 Chestnut Health Systems, 448 Wylie Dr, Normal, IL 61761 USA; 4 Research Triangle Institute, 3040 E. Cornwallis Rd, Research Triangle Park, NC 27709-2194 USA

**Keywords:** Substance use treatment, Adolescent, Program sustainment, Longitudinal analysis

## Abstract

**Background:**

Implementation support models are increasingly being used to enhance the delivery of evidence-based treatments (EBTs) in routine care settings. Little is known about the extent to which these models lead to continued EBT use after implementation support ends. Moreover, few empirical studies longitudinally examine the hypothesized factors associated with long-term psychosocial EBT use (i.e., sustainment). In an effort to address this gap, the current study examined sustainment of an EBT called the Adolescent-Community Reinforcement Approach (A-CRA) following the end of implementation support.

**Methods:**

Between 2006 and 2010, the Substance Abuse and Mental Health Services Administration awarded 3 years of A-CRA implementation support to 82 community-based organizations around the USA. The extent to which A-CRA was sustained following grant end and the hypothesized factors associated with EBT sustainment were collected using both retrospective and prospective data. We examined the extent to which 10 core treatment elements of A-CRA were sustained and the associations between the extent of A-CRA sustainment and hypothesized factors using a pattern-mixture longitudinal modeling approach.

**Results:**

Staff from 76 organizations participated in data collection for a 92.86% response rate. On average, about half of the 10 core treatment elements were sustained following the loss of implementation support. Factors that appeared most important to A-CRA sustainment included characteristics that were related to the outer setting (communication, funding, and partnerships), inner setting (political support, organizational capacity, and supervisor turnover rate), implementation support period (number of clinicians and supervisors certified and employed at support end and number of youth served), and staff perceptions of the intervention (implementation difficulty, relative advantage, and perceived success).

**Conclusions:**

Even with multiple years of implementation support, community-based organizations face challenges in sustaining EBT delivery over time. Consistent with implementation theories, multiple factors appear related with EBT sustainment, including the degree of implementation during the initial support period, as well as adequate funding, infrastructure support, and staff support following the end of funding.

## Background

Research suggests many evidence-based treatments (EBTs) are discontinued or rarely used following the end of initial implementation support [1, 2]. Moreover, the previous literature offers limited empirical evidence about the factors related to psychosocial therapy program sustainment in routine substance use disorder treatment settings [3]. Without effective EBT sustainment, investments in implementation are spent unwisely and public health impact is limited [4, 5]. Thus, there is a need to characterize the extent of EBT sustainment following the end of implementation support and to identify factors that are predictive of sustainment as they may serve as targets for sustainment strategies.

### Previous literature

In the past decade, there has been a growing body of evidence on measuring EBT sustainment and the hypothesized predictors [6]. However, recent reviews suggest that research within the health care field is underdeveloped [7, 8]. One issue that has resulted from this work is the diversity of definitions regarding how to assess program sustainment. Generally, it has been assessed by a simple self-reported dichotomous indicator of whether a program is still in operation or not following an initial funding period [6]. Although a dichotomous variable of whether a treatment was delivered or not may be appropriate for medication trials or to understand utilization rates (e.g., [9]), findings from systematic reviews emphasize the need to better capture the extent or quality of sustainment because partial sustainment may not lead to the same desired outcomes observed in research trials, especially in relation to more complex treatments such as psychotherapy. Moreover, the research to date suggests that “partial” sustainment over full sustainment is commonly found after implementation support ends [7, 8, 10].

Systematic literature reviews also suggest that program sustainment is an intricate process, and the determinants or predictors of sustainment are mixed and vary across health care domains. As this study was conceptualized, two major implementation frameworks were published, the Exploration, Practice, Implementation, and Sustainment model (EPIS; [11]) and the Consolidated Framework For Implementation Research (CFIR; [12]). These frameworks both suggest that characteristics related to the broader community environment, external to the organization (i.e., “outer” setting or context), and characteristics operating within the organization or “inner” setting or context, such as organizational leadership, climate and culture, adequate staffing, and organizational processes, that help support EBT implementation such as strategic planning are relevant. In addition, characteristics of individuals charged with implementing the intervention and characteristics of the intervention itself are important. In sum, these conceptual frameworks suggest the need to take into account multiple factors operating at the system, organization, and treatment provider level to understand EBT adoption, implementation, and sustainment [13].

Taking into account these conceptual framework perspectives, findings from recent empirical studies have been inconsistent [1] and limited. For example, the use of small samples are common (e.g., prospective data from 32 programs [14]; 11 systems operating in two states [15]), studies that have been limited to the examination of demonstration projects (e.g., [10]) rather than programs operated by established organizations, and some studies that have only examined a subset of hypothesized factors, such as inner context characteristics (e.g., [5, 16]) and few rigorous studies have been conducted that use longitudinal data and measure the quality or extent of EBT sustainment. Therefore, a continued need exists for comprehensive empirical studies assessing the extent or quality of sustainment and the numerous hypothesized factors that predict it. Moreover, there is also little information about EBT sustainment in relation to psychosocial treatments for substance use which is typically provided in community-based settings that are often low or under-resourced and experience high staff turnover [17–19]. The current study helps address these gaps by examining the extent to which an EBT is sustained and what implementation factors, including a comprehensive set of potential factors that span both the inner and outer setting along with implementation- and intervention-related characteristics, are associated with sustainment.

### Current study

Substance use disorder treatment and implementation support are largely funded in the USA through public sources [20], such as discretionary grants that enhance organizational capacity to utilize EBTs. In this study, we investigated one of the largest investments to date by the Substance Abuse and Mental Health Services Administration’s (SAMHSA) Center for Substance Abuse Treatment (CSAT) to address adolescent substance use disorders, by providing multiple year support to community-based organizations to implement the Adolescent-Community Reinforcement Approach (A-CRA). A-CRA has been found in five randomized controlled studies to yield positive outcomes across alcohol use, mental health, and social domains [21–25], and it is listed in SAMHSA’s National Registry of Evidence-based Programs and Practices (N-REPP). A-CRA supports youth and parents by enhancing family, social, educational, and/or vocational reinforcers to aid in recovery from substance use. As of January 2016, over 270 organizations received support to deliver A-CRA [26].

### A-CRA implementation support model

The discretionary grants that treatment organizations received from SAMHSA during its initial dissemination efforts included approximately $300,000 annually for 3 years, plus three and half days of in-person A-CRA training for up to five clinical supervisors and clinicians followed by ongoing telephone coaching calls. The training processes were designed based on effective methods for disseminating EBTs [27, 28] and are described in greater detail in Godley et al. [6]. For example, individual clinicians received numeric and narrative feedback specific to reviews of recorded therapy sessions based on a detailed coding manual from external A-CRA experts [29] until they demonstrated competence and then randomly thereafter. A supervisor training process was designed to train supervisors to become internal A-CRA experts and included requirements that supervisors were able to reliably rate recorded therapy sessions and conduct regular internal supervision.

### A-CRA sustainment

As with many EBT dissemination efforts, prior to the current study little was known about how effective the A-CRA implementation support effort was in promoting long-term use of A-CRA. We operationalized sustainment by assessing the extent to which core treatment elements were maintained following the end of the implementation support period and adequate organizational capacity to continue maintenance of these core elements was demonstrated. This definition is consistent with the recommendations from a recent systematic review [8], and this operationalization was also recently employed by Aarons and colleagues [15, 30]. The elements align with the definitions of implementation fidelity (e.g., [31, 32]) in that they included measurement of treatment dose, quality (as measured by staff knowledge, certification, access to high-quality training, and supervision), and penetration (i.e., proportion of staff trained and youth treated in A-CRA).

We attempted to capture factors related to external and internal support for A-CRA implementation and take into account assessments of the extent or quality of implementation during the funding period to capture how well organizations had built the capacity to continue to deliver A-CRA following the loss of implementation support. This approach is consistent with implementation theories that suggest that program sustainment only can occur within an organization that has first committed to adopting and implementing a treatment or practice [11]. In sum, the purpose of this study was to examine the extent of A-CRA sustainment following loss of federal funding among community-based organizations and identify what hypothesized implementation factors were empirically related to sustainment.

## Methods

### Study context

This project examined A-CRA sustainment among 82 community-based organizations that were awarded an implementation support grant by SAMHSA/CSAT between 2006 and 2010. During that period, there were four program cohorts called the “Assertive Adolescent Family Treatment” initiatives that encouraged the use of A-CRA as the treatment approach (e.g*.*, [33]). In addition, other SAMHSA funding opportunities were offered during this period including the “Juvenile Drug Court” and “Juvenile Drug Treatment Court,” the “Offender Reentry Project,” and the “Targeted Capacity Expansion” initiatives. For these initiatives, the community-based grantee was required to identify an EBT, and several of the funded organizations selected A-CRA and therefore were included in our study sample.

### Sample

#### Organizations

The study sample was composed of established treatment organizations located in the USA. To receive the implementation support grant, applicants were required to demonstrate substance use treatment operation for at least 2 years prior to the proposed project period and compliance with local licensing, accreditation, and certification requirements.

#### Staff

Clinical supervisors and clinicians from the grantee organizations were recruited to participate. We aimed to enroll at least two individuals from each program responsible for youth treatment who were familiar with A-CRA. Because clinical supervisors and clinicians were trained and certified at different levels as part of the implementation process, it was important to include them both in the study sample.

### Data sources

We used administrative data collected from organizations during their respective funding period to assess the implementation-related factors. We also collected three waves of interview and survey data approximately 9 months apart starting in Fall 2013 and ending in Spring 2015.

### Primary data collection procedures

We used an administrative dataset containing the contact information of staff who were trained during the 3-year implementation period. Consistent with effective tailored survey methods [34], the recruitment strategy employed multiple methods (i.e., mail, phone, and email) to introduce and remind potential participants about the study. Once the phone interviews were completed, participants were sent an email with instructions on how to access an online survey.

The staff from the grantee organizations were re-contacted approximately 9 months following their first interview (and 9 months after the second interview for the third wave of interviews) to complete another interview and survey describing their experience with delivering A-CRA. If a participant reported that their organization was no longer delivering A-CRA at the proceeding wave, the organizations’ status regarding A-CRA discontinuation was confirmed at the following interview wave.

### A-CRA sustainment

#### Overview

Based on A-CRA implementation indicators that were monitored during the implementation support period, we assessed A-CRA sustainment by examining how well each organization maintained the 10 core treatment elements (see Table 1 for details on the measurement and scoring of each element). As shown in this Table under the “Source” column, the elements were assessed using primarily information collected from interviews or surveys conducted with clinical supervisors and/or clinicians at each of the participating organizations. Regarding the clinical and supervisor certification elements, self-reported information was verified using training support records from the team responsible for A-CRA certification. Also, training agendas were collected and reviewed for appropriate content by A-CRA experts.

**Table 1:** Ten core treatment elements of A-CRA sustainment

Core A-CRA element	Source	Measurement	Scoring
1. Clinical knowledge of A-CRA	Supervisor and clinician survey	10 multiple-choice items that were derived from the A-CRA treatment manual which covered the theoretical underpinnings and the content of selected A-CRA procedures [53]. During the implementation phase, clinicians and supervisors were required to demonstrate proficiency on these items in order to obtain A-CRA certification.	Each item included five response options, and answers were coded as correct (scored as a “1”) or incorrect (scored as a “0”) for a total score range from 0 to 10. In organizations where more than one respondent delivered A-CRA, scores were averaged across respondents.
2. A-CRA dosage	Clinician interview	One open-ended item that asked how many A-CRA treatment sessions clients were told that they would receive	If all clinicians at an organization endorsed a value within the treatment developers’ recommended range of sessions (i.e., 11–15 sessions), the organization received a score of “1;” otherwise, the organization scored “0”.
3. Clinical staff penetration	Supervisor interview and verification with certification documentation	Supervisors were asked (a) how many clinicians were on staff in the adolescent substance use treatment program and (b) the number and name of those clinicians who were certified to deliver A-CRA	Responses were verified using the treatment developer records that documented who had successfully completed the certification process. Each organization received a score from 0 to 1 based on the proportion of clinicians in the youth treatment program that were certified to deliver A-CRA.
4. Presence of certified A-CRA supervisor(s)	Supervisor interview and verification with certification documentation	Supervisors were asked whether their organization currently had any A-CRA-certified clinical supervisor(s) within their adolescent substance use treatment program	Responses were verified using the treatment developer records that documented who had successfully completed the supervision certification process. Each organization received a score of “1” for having an A-CRA-certified clinical supervisor on staff at the time of the interview or a “0” if no current staff were certified to provide A-CRA clinical supervision.
5. Recommended frequency of clinical supervision	Clinician interview	Clinicians were asked how often they received clinical supervision. Bi-weekly clinical supervision is emphasized as part of the A-CRA training protocol [20].	Organizations where clinicians reported receiving clinical supervision every 2 weeks or more were coded a “1” and organizations where clinicians reported receiving clinical supervision less frequently were coded a “0.” If more than one clinician responded per organization, the responses were averaged for an organization-level score.
6. Recommended clinical supervision content	Clinician interview	Six items based on the rating manual used for coding-recorded supervision sessions during the supervision certification process [54] were used. Example items were, “My supervisor reviews my A-CRA case review report” with “yes” and “no” as response options and “My supervisor role plays the correct way to do a procedure.”	Responses to the six items were scored correct (coded as “1”) or incorrect (coded as “0”). Two items were weighted more due to their relative importance and the average proportion correct was used for a range of 0–1.5. If more than one clinician responded per organization, the responses were averaged for an organization-level score.
7. Review and feedback on treatment sessions	Clinician interview	Clinicians were asked whether their clinical supervisor reviewed recorded treatment sessions and (a) told the clinician what they had done well and (b) gave suggestions about how the clinician could improve treatment delivery. These items were based on the guidelines in the A-CRA treatment manual.	Responses were scored “0” if clinicians reported that their clinical supervisors did not review sessions, “2” if the sessions were reviewed and clinicians were told they had done well, or “4” if session review also included suggestions on how treatment delivery could be improved. If more than one clinician responded per organization, the responses were averaged for an organization-level score.
8. Clinical supervisors’ knowledge of A-CRA training and certification process	Supervisor interview	Supervisors who had reported training staff in the A-CRA model were asked 15 true or false statements about the certification process. These statements were drawn from a list of requirements of the A-CRA certification process and outlined in the A-CRA coding manual [29] that supervisors were required to master as part of the A-CRA treatment certification process. An example question was, “When I decide to pass a clinician on a procedure, it is based on ratings of 1 or more on every component of a procedure.” Clinical supervisors were also asked an open-ended interview question about how many therapy session recordings were needed to reach basic clinical certification.	Organizations where supervisor(s) correctly answered the recording question (i.e., the correct answer was 8 or more recordings) received a “1” and organizations where respondents incorrectly answered this question received a “0.” The score on the certification process questions and recordings question were combined by taking the average of these two measures to form the score on this element. In organizations with more than clinical supervisor, the percentage correct was averaged to create an organization-level value that ranged from 0 to 100%.
9. On-site A-CRA training quality	Supervisor interview and documentation	Supervisors who reported providing A-CRA training at their organization were asked to submit a training agenda. Submission of a training agenda was one of the required elements for the A-CRA Clinical Supervisor Certification process and supervisors were provided feedback until their agendas met specified criteria	Submitted agendas were rated by a member of the A-CRA implementation support team for its coverage of key A-CRA training aspects. Scores ranged from 0 to 1 representing the proportion of A-CRA training aspects covered (e.g., inclusion of half of the training elements would be scored 0.5).
10. Youth penetration	Supervisor interview	Supervisors were asked (a) how many youth clients had received substance use treatment at their organization in the past 6 months and (b) how many youth received A-CRA during that same period.	A proportion of youth clients receiving A-CRA (of those receiving any substance use treatment) was calculated for each organization (range 0–1).

### Sustainment period and self-reported status

We used administrative data about when the organization stopped receiving the last SAMHSA/CSAT funding (i.e., grant end date) to calculate the time between the funding end date and the study data collection dates. We utilized the number of months since the last grant end date to characterize the time since funding loss and used it as a control variable in the main regression analyses.

Participants were asked at the beginning of the interview whether their organization was currently delivering A-CRA and if the organization had stopped using A-CRA, to report when it had stopped being delivered. A dichotomous indicator of whether the organization was sustaining A-CRA or not was calculated and used to address missing data (see Analytic Plan).

### Implementation characteristics

#### Outer setting characteristics

We used several subscales of the Program Sustainability Assessment Tool (PSAT) [35, 36] to assess outer and inner setting factors. Each subscale consisted of five items and the alphas among our sample ranged from 0.84 to 0.95. Responses ranged from “to little or no extent” (scored as a “1”) to “to a great extent” (scored as a “7”), we summed the responses (for a range of 5–35) and calculated the mean values for each scale. The following subscales were used: communications to capture strategic communication with stakeholders and the public, funding stability to assess whether staff reported that the A-CRA program had a consistent and stable funding source, partnerships to measure community support for the program and political support to gauge political environments (e.g., “Political champions advocate for the program”). Of note, some of subscales assess both outer and internal contextual characteristics or are ambiguous in regard to setting, but we classified them here for simplicity.

#### Inner setting characteristics

*Structural factors*: We examined the comprehensiveness of the organizations, by evaluating a count of services offered at the organization using a survey question from the National Survey of Substance Abuse Treatment Services [37]. The range on this variable was from 0 to 17. We also asked what the primary focus of the organization was with the following response options: substance use treatment services, mental health services, a mix of mental health and substance use services (neither is primary), general health care, and others. A binary variable was used that indicated whether staff reported that substance use treatment was the primary service (coded “1”) as compared to all other options (coded “0”). *Staffing characteristics*: We assessed current staffing characteristics that may help explain the organization’s capacity to sustain A-CRA. More specifically, we calculated clinical supervisor and clinician turnover rates based on interview questions with supervisors for the past 6 months. Given that treatment implementation may be influenced by the number of staff available, we also include a variable of the client-to-staff ratio that was calculated based on the supervisor reports of the number of youth served and number of clinicians serving youth in the past 6 months. *Staff attributes*: The Organizational Readiness for Change (ORC) scale [38] operationalizes staff attributes using the following subscales: adaptability, efficacy, and influence. We also asked both supervisors and clinicians about organizational climate using items from the ORC scale [38] that included the following subdomains: autonomy, cohesion, communication, mission, and stress. Responses from both ORC scales were scored on a five-point scale from “strongly disagree” (coded as a “1”) to “strongly agree” (coded as a “5”). Responses were summed and averaged across each of the subscales in the organizational climate domain, and then, the subscale values were averaged and multiplied by 10 to create a value between 10 and 50. *Implementation leadership*: We included the 12-item version of the Implementation Leadership Scale [39]. Clinician responses were recorded on a five-point scale from 1 indicating “strongly disagree” to 5 indicating “strongly agree” then summed and averaged. We assessed organizational resources and support to effectively manage A-CRA using the organizational capacity subscale of the PSAT that was asked of both clinicians and supervisors. *Strategic planning* for sustainability was assessed using the corresponding PSAT subscale.

#### Implementation-related characteristics

The extent to which an organization has consistently implemented the intervention during the funding period is likely to influence how well it can be sustained post the initial support period [11, 40]. In order to examine this, we utilized the data collected during the funding period that included (1) the number of adolescents that received A-CRA, (2) the number of clinical supervisors certified in A-CRA and still employed at each organization at grant end, and (3) the number of clinicians certified in A-CRA at each organization and still employed at the end of the grant period.

#### Perceptions of the intervention

Several theories suggest that perceptions of a particular innovation’s ease of use and benefit over alternative options will influence its adoption, use, and presumably longer-term sustainment [11, 12, 41, 42]. We included assessments of staff perceptions of A-CRA’s complexity and relative advantage from Steckler et al. [43] (alphas = 0.88 and 0.83, respectively). We included staff perceptions of implementation difficulty and perceived success using five-item scales developed from O’Loughlin et al. [44] (alphas = 0.57 and 0.91, respectively). All of these survey items had response options on a five-point scale ranging from “strongly disagree” (scored as a “1”) to “strongly agree” (scored as a “5”) for a summed score range of 4–20 (for the relative advantage scale) or 5–25 (for the complexity, difficulty, and success scales).

### Analytic plan

First, data collected from staff was aggregated to the organization by wave level. The hypothesized outer setting, inner setting, and intervention-related variables were constructed using assessments collected from what we considered the “critical period”, that is, the first 18 months following the end of the implementation support period.

Prior to the longitudinal analyses, each of the 10 elements was standardized to 0–1 and summed for a score ranging from 0 to 10 at the site by wave level. Missing data in elements was imputed by using the means of observed data in each study wave and by self-reported sustainment status. The summed score is our main sustainment outcome in statistical analysis.

As planned [45], we conducted exploratory analysis to find potential clustering of sustainment trajectories. The organizations were grouped into two sustainment patterns according to the cluster analysis. The first pattern is consisted of organizations which reported stopping A-CRA delivery within 12 months at the end of the funding period. The second pattern is consisted of organizations which reported sustaining A-CRA longer than 12 months in the post-funding period (i.e., either stopped A-CRA after the 12th month in the post-funding period or continued sustaining A-CRA without observed stoppage). Since organizations in the first pattern had a shorter observation period (no more than two waves) than organizations in the second pattern, it is necessary to adjust for the mixing patterns in order to unbiasedly estimate the effects of predictors [46, 47]. Lastly, we fitted a set of pattern-mixture longitudinal models to estimate the marginal relationship between the sustainment outcome and each predictor separately. In these pattern-mixture models, we also controlled for time after the funding ended (in months). We used random effects to account for intra-site correlations among the longitudinal measures of sustainment scores. The mixed-effect model also allows for unsynchronized measurement times so that we could consistently estimate the marginal association. To account for multiple comparisons, we applied the step-up methods to adjust *p* values to control the false discovery rate at the .05 [48].

## Results

### Sample characteristics

One hundred and sixty-nine respondents (i.e., 33% who classified themselves as supervisors, 41% who classified themselves as clinicians, and 26% who classified themselves as both supervisions and clinicians) from 78 organizations participated in the data collection for a 92.86% response rate at the site level. The range in time since funding loss ranged from 1 to 63 months for the 78 organizations. Table 2 shows the distribution of organizations by the different funding sources. While most organizations received one grant (*n* = 63), 15 organizations received multiple rounds of funding, including four organizations who received three or more awards. Most of these multi-funded sites (i.e., 12 of the 15 organizations) were in the second mixture pattern (i.e., demonstrating sustainment longer than 12 months in the post-funding period).

**Table 2:** The number of participating organizations by the different funding mechanisms

	None	AAFT1	AAFT2	AAFT3	AAFT4	AAFT1 and AAFT3	AAFT1 and AAFT4	AAFT2 and AAFT3	AAFT2 and AAFT4	AAFT3 and AAFT4	AAFT1, AAFT3, and AAFT4	Totals
JDC	3									1		4
JTDC	5	1								2		8
ORP	6										1	7
TCE	1	1										2
None		4	15	6	23	3	4	1	1			57
Totals	15	6	15	6	23	3	4	1	1	3	1	78

“None” in the column means no funding from the AAFT initiatives; “None” in the row means no JDC, JTDC, ORP, or TCE initiatives

*AAFT* Assertive Adolescent Family Treatment, *JDC* Juvenile Drug Court, *JDTC* Juvenile Drug Treatment Court, *ORP* Offender Reentry Program, *OJJDP* Office of Juvenile Justice & Delinquency Prevention, *TCE* Targeted Capacity Expansion

The average age of participants was 41.74 years (SD = 11.57). Sixty-five percent of the sample was female. Thirty-three percent of the sample reported that they were of Hispanic origin. The racial composition reported by respondents was 70% White; 13% Black/African-American; 5% Native American or Alaskan; 2% Asian, Native Hawaiian, or other Pacific Islander; 1% more than one race; and 10% did not endorse a race. Sixty-five percent of the sample reported a master’s level of education, 22% reported a bachelor’s level education, 5% reported an associate’s level of education, 4% reported a doctoral degree, and 4% reported some college. The average number years of substance use disorder counseling experience among the sample was 9.44 (SD = 7.84).

### Extent of A-CRA sustainment

Descriptive statistics for the 10 elements that comprise the sustainment outcome are reported in Table 3. Although clinical knowledge was generally good (i.e., overall percentage correct was 70%) and most organizations (77%) had a certified A-CRA supervisor on the staff, many of the other elements were not sustained at recommended levels. For example, less than half of the clinicians on the staff were A-CRA certified and less than half of the organizations reported delivering the recommended dosage of A-CRA. Consequently, on average, about 40% of adolescents were receiving A-CRA at these organizations. Elements related to clinical supervision, including frequency and content were lower than recommended. Supervisor knowledge of the A-CRA training and certification process was fairly low (30%), and onsite training quality was especially low with on average only 15% of the desired features included in the training agendas. Organizations that sustained A-CRA more than 12 months had higher values on 7 of the 10 elements. Elements related to clinical supervision frequency and content were similar or higher in organizations in the sustaining a year or less group.

**Table 3:** Descriptive statistics of the 10 core A-CRA elements

Element (range)	All		Mixture pattern ≦12 months		Mixture pattern >12 months	
	Mean	SD	Mean	SD	Mean	SD
1. Clinical knowledge of A-CRA (0–1)	0.70	0.21	0.68	0.23	0.72	0.18
2. A-CRA dosage (0–1)	0.44	0.50	0.33	0.49	0.50	0.51
3. Clinical staff penetration (0–1)	0.35	0.40	0.22	0.37	0.49	0.38
4. Presence of certified A-CRA supervisor(s) (0–1)	0.77	0.42	0.61	0.49	0.92	0.27
5. Recommended frequency of clinical supervision (0–1)	0.57	0.50	0.71	0.46	0.45	0.51
6. Recommended clinical supervision content (0–1.5)	1.06	0.34	1.11	0.39	1.02	0.30
7. Review and feedback on treatment sessions (0–4)	2.12	1.97	2.71	1.86	1.70	1.98
8. Clinical supervisors’ knowledge of A-CRA training and certification process (0–1)	0.30	0.33	0.24	0.31	0.36	0.34
9. On-site A-CRA training quality (0–1)	0.15	0.24	0.00	0.00	0.15	0.24
10. Youth penetration (0–1)	0.40	0.40	0.20	0.35	0.58	0.36

### Factors associated with A-CRA sustainment

Mean values or proportions on the hypothesized factors and the sustainment outcome are presented in Table 4. The table shows the distributions by the two groupings, organizations that sustained A-CRA less than 12 months and organizations that sustained A-CRA 12-months or longer. In general, these mean values and proportions show that organizations with longer periods of sustainment have more support in terms of the outer and inner setting and implementation- and intervention-related characteristics.

**Table 4:** Descriptive statistics on the hypothesized factors and outcome by sustainment time

	Mixture pattern ≦12 months		Mixture pattern >12 months	
	(*n* = 40)		(*N* = 38)	
	Mean	SD	Mean	SD
External setting				
Communications	22.98	4.97	22.54	6.58
Funding stability	14.92	4.89	19.54	5.85
Partnerships	19.67	6.11	20.74	6.23
Political support	21.19	5.21	24.05	5.62
Inner setting				
Clinician turnover rate	0.37	0.41	0.36	0.85
Implementation leadership	46.75	8.82	50.47	4.60
Organizational capacity	24.61	6.61	25.14	5.60
Strategic planning	20.26	5.25	22.03	5.34
ORC Scale: org climate	36.40	4.35	37.85	2.48
ORC Scale: staff attributes	41.03	4.72	42.49	2.42
Agency focus	0.35	0.44	0.54	0.40
No. of services offered	12.00	2.77	12.81	2.08
Client-staff ratio	5.54	7.31	10.26	8.96
Supervisor turnover rate	0.48	0.77	0.24	0.45
Implementation-related				
No. of clinicians certified/employed at grant end	1.47	1.20	2.71	1.47
No. of supervisors certified/employed at grant end	0.84	0.72	1.00	0.77
No. of youth served during grant period	91.92	44.40	144.21	109.83
Perceptions of the intervention				
Complexity	7.21	2.32	5.84	1.59
Implementation difficulty	16.14	3.13	14.94	2.45
Perceived success	20.38	3.33	20.88	2.42
Relative advantage	15.90	2.97	16.61	1.79
Outcome				
Sustainment score	5.53	1.24	6.37	1.25

Table 5 presents the results from the marginal regression analyses including coefficients and *p* values accounting for a false discovery rate at the level of .05. The results show that higher ratings on the PSAT scales related to communications, funding stability, partnerships, political support (i.e., primarily external setting factors), and organizational capacity as well as strategic planning (i.e., internal setting factors) were associated with greater levels of A-CRA sustainment. The results also show that higher rates of clinical supervisor turnover were associated with lower levels of A-CRA sustainment. Performance during the implementation support period, as assessed by the number of youth who received A-CRA and number of clinical supervisors and clinicians that had been certified and were still employed at the organization at the end of the implementation support period were strongly associated with the extent to which A-CRA was sustained. Finally, perceptions of A-CRA by clinical staff were also associated with the extent to which A-CRA was sustained. Staff who perceived A-CRA as rather easy to implement were more likely to be employed at sites that sustained higher levels of A-CRA. Also, perceptions that A-CRA was an efficacious treatment and better than other available treatments were associated with organizations sustaining higher levels of A-CRA sustainment.

**Table 5:** Results from the marginal regression analyses predicting the extent of sustainment

Predictors	Estimate	SE	*t* value	*p* value
External setting				
Communications	0.05	0.02	2.98	0.005*
Funding stability	0.05	0.02	2.97	0.006*
Partnerships	0.05	0.02	2.99	0.005*
Inner setting				
Clinician turnover rate	0.43	0.53	0.81	0.425
Implementation leadership	0.01	0.01	1.23	0.226
Organizational capacity	0.07	0.02	4.39	<0.001*
Political support	0.06	0.02	3.16	0.003*
Strategic planning	0.05	0.02	2.35	0.025*
ORC Scale: org climate	0.04	0.02	1.67	0.106
ORC Scale: staff attributes	0.04	0.03	1.33	0.195
Agency focus	−0.04	0.30	−0.13	0.899
No. of services offered	0.03	0.06	0.44	0.666
Client-staff ratio	0.00	0.01	0.43	0.670
Supervisor turnover rate	−1.21	0.35	−3.46	0.003*
Implementation-related				
No. of clinicians certified/employed at grant end	0.32	0.11	2.91	0.006*
No. of supervisors certified/employed at grant end	0.55	0.20	2.73	0.010*
No. of youth served during grant period	0.01	0.01	2.61	0.014*
Perceptions of the intervention				
Complexity	−0.04	0.07	−0.63	0.536
Implementation difficulty	−0.14	0.04	−3.30	0.002*
Perceived success	0.14	0.05	2.61	0.014*
Relative advantage	0.17	0.06	3.03	0.005*

*Statistically significant controlling for false discovery rate <0.05

## Discussion

In this study, we explored the extent to which an evidence-based treatment to address adolescent substance use (i.e., A-CRA) was sustained following the end of federally funded implementation support grants to 78 community-based organizations. Using recent recommended approaches, we operationalized A-CRA sustainment by considering the extent to which organizations maintained core treatment elements following the end of the implementation support period and adequate organizational capacity to continue maintenance of these core elements was demonstrated. Our results showed that following the loss of implementation support, partial sustainment of the core elements was frequently found. These findings are comparable to previous studies that have documented that the quality of program delivery tends to decline after implementation support ends (e.g., [10, 15, 49]).

Examining the scores on the 10 core elements helps to explain the capacities that may facilitate and hinder organizations to continue A-CRA. It seems that organizations that continue A-CRA beyond 12 months were more likely to have a certified A-CRA clinical supervisor on staff and a higher proportion of A-CRA-certified clinicians than organizations with shorter sustainment periods. However, staff from organizations that reported sustaining A-CRA for a year or less reported higher scores on clinical supervision frequency and content elements compared to organizations that reported sustaining A-CRA for longer periods. This pattern suggests that organizations that maintain A-CRA delivery may be doing so with lower supervision quality which may have implications for treatment outcomes. The results also suggest that organizations that maintain higher clinical supervision intensity that is aligned with the A-CRA content are less likely to continue A-CRA than organizations that employ supervision with lower intensity and aligned content.

Our findings are in line with others which have revealed that “partial” sustainment over full sustainment is commonly found after implementation support ends [7, 8, 10]. It is the first study of its kind to examine sustainment for an adolescent substance use EBT, so it is not possible to judge whether the level of sustainment is better or worse for this type of intervention. Additionally, the measures used for sustainment are unique to the EBT. For example, Bond et al. [1] reported a 96% sustainment rate of an Individual Placement and Support (IPS) learning community approach based on an operational definition of sustainment that included whether or not a program continued to employ staff, maintained an active client caseload, provided direct services, and adhered to core principles. The latter rate is impressive, but assessing adherence to a set of core principles appears to be a more generalized measure of sustainment than adherence to very specific treatment procedures. Since EBPs have different characteristics, it is important as the study of sustainment increases to make comparisons across similar types of programs or treatments. Further work is needed to develop more standardized measures of sustainment. Differences between the sustainment rates reported here and those in the Bond et al. study may also be due to a broader availability of funding for the IPS services which included state and county agencies rather than one-time federal grants to community-based organizations. Such organizations may embrace the opportunity for federal funding so that they can hire additional staff and serve more individuals even if they know it will be difficult to sustain a given practice after the time limited funding ends. Sustainment of A-CRA or other EBTs that address substance use may improve with increased support, including reimbursement for both treatment delivery and clinical supervision to aid in the achievement of the outcomes found in the research studies where adequate time for quality clinical supervision is provided.

When we examined what factors were associated with the extent of A-CRA sustainment, we found that, consistent with implementation frameworks, characteristics of both the outer and inner settings were related to sustainment. Also, how well the organization was positioned to sustain A-CRA at the end of the implementation support period, based on their performance and capacity at grant end, was also found to be a strong predictor of the extent of sustainment. Finally, staff perceptions of A-CRA were also found to be strongly associated with the extent of sustainment.

These findings are consistent with previous theories that suggest that multiple factors including those that are external to the organization as well as within an organization are key to EBT sustainment [12, 41, 50]. Our study findings are also relatively consistent with the emerging empirical literature on EBT sustainment. For example, Hodge et al. [5] examined factors related to EBT sustainment among a large sample of “Triple P” (i.e., a parenting prevention program) providers. Similarly, they found the importance of organization supports (e.g., clinical supervision) and intervention-related characteristics (e.g., perceptions of the implementation ease). However, their study was limited in that external contextual factors were not examined. Peterson et al. [16] also examined the inner context or setting factors related to the sustainment of five mental health EBTs over an 8-year period. Unlike our study, they did not find implementation quality predictive of long-term sustainment; however, like our study, staff turnover appeared important. Tibbits et al. [14] examined the sustainment of crime and delinquency programming in school settings up to 3 years post-funding. The investigators found that leadership support, overall school support, adequate staffing, financial stability planning, and aligning the intervention with the setting (i.e., “fit”) was related to self-reported sustainment among a small sample of programs.

The study findings build on the results reported from a previous study of a subset of the same organizations (*n* = 68) using a binary measure of self-reported A-CRA sustainment [51]. In the previous study, we also found support for the association between external setting, inner setting, implementation-related and intervention-related factors, and A-CRA sustainment. However, a few variables that were found related to A-CRA sustainment in that study were not found to be significantly associated with the extent of sustainment found in this study (and vice versa). More specifically, in the previous study, the type of agency and perceptions of A-CRA’s complexity appeared important and such external setting factors as communications, partnerships, and internal factors such as organizational capacity were not found to be statistically significant. A key difference between the two studies is in the outcome: the previous study examined whether staff reported A-CRA was continued at the site and the current study uses the extent to which A-CRA was delivered using a more comprehensive measure of the extent of sustainment. These distinctions are important because continuing A-CRA without regard to the core elements may not lead to the same quality in delivery and thus outcomes achieved.

In sum, this study demonstrates that many characteristics cited by implementation theories or frameworks are critical to the quality in which an EBT for adolescent substance use disorders is supported in community-based substance use treatment organizations after implementation support ends. These findings point to the relevance of paying close attention to both internal and external supports to an organization to assist with EBT sustainment. Internal supports include maintaining qualified clinicians and supervisors for psychosocial treatments. External supports include the availability of other sources of funding and support in the external environment when implementation funding is ended.

It is also relevant to note that many theorized constructs were not found to be statistically significantly related to A-CRA sustainment, including clinical turnover rate, perceptions of leadership support, staff attributes, perceptions of A-CRA complexity, and characteristics of the organization including structural factors (focus, comprehensiveness), and climate. We think that clinician turnover rate may have been buffered by the support provided to supervisors to train and certify locally. In support of this, supervisor turnover was significantly related to sustainment. It appeared that external factors and how well the organization was prepared at grant end as demonstrated by certified staff and experience treating youth, along with organizational factors such as capacity, political support, and strategic planning were more critical to sustaining A-CRA than these other inner setting characteristics. Thus, the size or mission of the organization is less important than ongoing funding and support outside the agency coupled with well-trained staff that perceive the intervention positively.

We address several limitations to previous research on health care program sustainment. For example, few studies have used conceptual implementation frameworks to inform their work. This study was developed taking into account several existing conceptual approaches to program implementation and sustainment, and therefore, we assessed factors both external to and internal to the organization along with intervention-specific components to sustainment. Also, few studies have employed longitudinal, prospective methods. Given the extensive data collected during the implementation period and the longitudinal nature of this study, we were able to examine several variables prospectively to predict the extent of A-CRA sustainment. Moreover, we assessed the extent of sustainment by assessing the level of 10 core treatment elements rather than relying on a dichotomous measure of whether staff reported that their organization continued to deliver A-CRA. In sum, this study addresses many important gaps in previous research.

### Limitations

Some limitations to this study are important to acknowledge. First, this study has a relatively small sample. We targeted the entire population of organizations that were funded and achieved over a 90% response rate; however, to study the multitude of hypothesized factors and the potential interaction effects, a larger sample would be required; this is a commonly noted challenge in implementation research where the main analyses are often conducted at the organizational level rather than at the patient level [32]. Due to the sample size, we were unable to reliably estimate the effects using all of the hypothesized predictors in one model; therefore, our findings cannot address questions about the relative impact of the different hypothesized predictors compared to one another on the extent of sustainment. For example, we cannot address whether external factors are more or less important than staff experience with or perceptions of A-CRA. Also relevant, the CFIR contains 39 domains, and we did not examine all of these. Moreover, we were not able to examine clinical program outcomes and whether the extent of sustainment limits the impact of A-CRA on youth substance use and related outcomes. We also did not verify some of the A-CRA elements (e.g., frequency and content of supervision); staff may have endorsed higher levels than provided on some of these activities. Future research is warranted on whether varying levels of A-CRA sustainment observed following funding loss influences the effectiveness of A-CRA.

## Conclusions

Despite the recent development of several evidence-based treatment (EBT) programs for substance use, less than half of adolescents are positively discharged from treatment [52], suggesting the need for the practice of effective treatments. Although the A-CRA has demonstrated effectiveness, we found that following an implementation support period, community-based substance use treatment organizations found longer term sustainment challenging. Successful implementation during the initial funding period appeared an important factor to longer term sustainment. This finding demonstrates the need to closely monitor and support implementation during the funding phase. Other important external factors were funding stability and community and political support for the treatment. Critical organizational factors included adequate supervisor staffing, organizational capacities, and positive perceptions about the treatment by clinical staff. As government and other entities consider support for the implementation of EBTs, it is important for them to consider what types of settings, infrastructures, and organizational factors should be present during the selection process to ensure investments are well spent.

## Abbreviations

A-CRA: Adolescent community reinforcement approach
CFIR: Consolidated framework of implementation research
CSAT: Center for substance abuse treatment
EBT: Evidence-based treatment
EPIS: Exploration, Practice, Implementation, and Sustainment
IPS: Individual placement and support
M: Mean
ORC: Organizational readiness for change
PSAT: Program sustainability assessment tool
SAMHSA: Substance abuse and mental health services administration
SD: Standard deviation
SE: Standard error
